# The effect of tooth cusp morphology and grinding direction on TMJ loading during bruxism

**DOI:** 10.3389/fphys.2022.964930

**Published:** 2022-09-15

**Authors:** Benedikt Sagl, Martina Schmid-Schwap, Eva Piehslinger, Xiaohui Rausch-Fan, Ian Stavness

**Affiliations:** ^1^ Center of Clinical Research, University Clinic of Dentistry, Medical University of Vienna, Vienna, Austria; ^2^ Division of Prosthodontics, University Clinic of Dentistry, Medical University of Vienna, Vienna, Austria; ^3^ Department of Computer Science, University of Saskatchewan, Saskatoon, SK, Canada

**Keywords:** bruxism biomechanics, computational biomechanics, bruxism, temporomandibular joint, temporomandibular joint disorder, mediotrusive interference, temporomandibular joint biomechanics

## Abstract

Increased mechanical loading of the temporomandibular joint (TMJ) is often connected with the onset and progression of temporomandibular joint disorders (TMD). The potential role of occlusal factors and sleep bruxism in the onset of TMD are a highly debated topic in literature, but ethical considerations limit *in vivo* examinations of this problem. The study aims to use an innovative *in silico* modeling approach to thoroughly investigate the connection between morphological parameters, bruxing direction and TMJ stress. A forward-dynamics tracking approach was used to simulate laterotrusive and mediotrusive tooth grinding for 3 tooth positions, 5 lateral inclination angles, 5 sagittal tilt angles and 3 force levels, giving a total of 450 simulations. Muscle activation patterns, TMJ disc von Mises stress as well as correlations between mean muscle activations and TMJ disc stress are reported. Computed muscle activation patterns agree well with previous literature. The results suggest that tooth inclination and grinding position, to a smaller degree, have an effect on TMJ loading. Mediotrusive bruxing computed higher loads compared to laterotrusive simulations. The strongest correlation was found for TMJ stress and mean activation of the superficial masseter. Overall, our results provide *in silico* evidence that TMJ disc stress is related to tooth morphology.

## Introduction

The masticatory region is one of the most complex human joint systems, which makes detailed research into its biomechanics difficult. Literature commonly connects increased temporomandibular joint (TMJ) loading with temporomandibular joint disorders (TMD), but ethical restrictions on *in vivo* investigations of the joint’s biomechanics, caused by its small size and intricate morphology, make detailed investigations of this connection problematic (Detamore and Athanasiou, 2003; Ingawalé and Goswami, 2009). Examples of frequently debated topics in dentistry include the effect of occlusal factors on TMJ loading (Hattori et al., 2003; Huang et al., 2006; Raphael et al., 2012; Wohlberg et al., 2012; Tokiwa et al., 2014) as well as the role of sleep bruxism in the onset of TMD (Manfredini et al., 2014; Manfredini and Lobbezoo, 2010). Historically, bruxism was defined as parafunctional, unconscious grinding and clenching of teeth (Ferro et al., 2017). A newer expert consensus definition describes bruxism as a motor behavior, instead of a parafunction, additionally includes thrusting of the mandible and emphasizes the distinction between awake and sleep bruxism (Lobbezoo et al., 2018; Manfredini et al., 2021).

Even when casting aside the ethical concerns of *in vivo* investigations of TMJ loading, there are many morphological variables for each of the 32 teeth, which make detailed studies into specific occlusal factors virtually impossible, since a very large study cohort would be necessary to account for the multiple occlusal variabilities present between patients. Moreover, recording muscle excitation patterns of the jaw region through electromyography (EMG) is complex compared to other joint systems. This is caused by the small size and large number of muscles in the region, which partly overlap each other and are consequently not easily accessible using surface electrodes. Hence, EMG of the masticatory region is generally solely collected for the masseter and temporalis muscles and potentially for the submental region as a whole (Castroflorio et al., 2008; Lavigne et al., 2005). The temporalis and masseter muscle are important for closing force creation, but the various tooth grinding movements performed during bruxing will facilitate combinations of different additional muscles to guide the mandible along the relevant tooth facet while applying closing force. In turn, recruitment of different patterns of muscle activation for tooth grinding movements subject to different occlusal factors is likely to create differences in TMJ disc stress.

Computer methods, such as musculoskeletal modeling (Andersen et al., 2017) and finite-element simulation (Commisso et al., 2014; Ortún-Terrazas et al., 2022) have become a common tool in craniofacial and dental research. For the analysis of TMJ loading during bruxism, *in silico* investigations can help to solve the above mentioned problems of *in vivo* investigations. They can easily be used to investigate the effect of a single variable (e.g. bruxing position), while keeping occlusal and TMJ relations otherwise untouched. Moreover, tracking simulation approaches enable the computation of muscle excitation patterns, without the requirement of EMG acquisition for all muscles. While multiple *in silico* investigations of bruxism exist, they mostly concentrated on the effect of static bruxing tasks on TMJ loading (Nagahara et al., 1999; Mori et al., 2010; Hattori-Hara et al., 2014; Wu et al., 2019). For instance, Commisso et al. (2014) examined the effect of cyclic and sustained loading, mimicking tooth clenching and pressing, on the TMJ disc (Commisso et al., 2014). Their results suggest that sustained clenching can induce shear stresses that are high enough to potentially damage the TMJ disc. While these previous *in silico* investigations of bruxing offer important information on the potential effect of static bruxing tasks on TMD development, many bruxing movements have a dynamic component and it is reasonable to expect that the combination of high closing forces and condylar motion might create even higher TMJ loads.

Most recent literature negates a direct link between sleep bruxism and increased myofascial pain (Castroflorio et al., 2008; Raphael et al., 2012), but to the best of our knowledge no study on the potential connection between sleep bruxism and increased mechanical TMJ loading exists. Consequently, the connection between occlusal factors and mechanical TMJ loading remains possible through biomechanical reasoning. A previous *in silico* investigation of dynamic, laterotrusive (LT) bruxing using two bruxing positions and 6 different lateral inclinations showed that both bruxing position and facet inclination influence TMJ loading, with inclination being of special interest (Sagl et al., 2022). These results were limited to only 12 simulation conditions and solely focussed on LT bruxing, but still supported the idea of a potential connection of tooth morphology and TMJ loading.

Another factor that is often discussed in relation to occlusion and TMD are mediotrusive (MT) interferences. The 9th Edition of the Glossary of Prosthodontic Terms defined occlusal interference as “any tooth contact that inhibits the remaining occluding surfaces from achieving stable and harmonious contacts” or “any undesirable occlusal contact” (Ferro et al., 2017). The connection between TMD and MT interferences is a highly contested topic within literature. On the one hand increased signs and symptoms in TMD patients with artificially placed MT interferences were reported (Le Bell et al., 2002) and presence of MT interferences had a statistically significant correlation with TMD diagnosis (Haralur, 2013). On the other hand, other studies found no clinically relevant connection between occlusal factors and joint pain or sounds (Aboalnaga et al., 2019; Stone et al., 2017). Recently, a consensus statement on MT occlusal contacts stated that “MT interferences may alter the biomechanics of mandibular function. In the presence of repeated high loads this can possibly lead to pathophysiology of the temporomandibular joint and associated muscle structures” (Walton and Layton, 2021). Accordingly, the investigation of MT interferences during bruxing is important to better understand the potential connection of TMD and bruxism.

Overall, the aim of this study was to investigate the effect of morphological parameters of the teeth as well as tooth grinding direction, with unilateral tooth contact, on TMJ loading. We report muscle activation patterns and TMJ von Mises stress, using our previously developed high-resolution model of the masticatory region and forward-dynamics tracking approach. A total of 450 simulations was computed for this investigation. The presented study used state-of-the-art computational methods for an exhaustive, *in-silico* investigation of the effect of bruxing cusp morphology and tooth grinding direction on TMJ loading. The results shed new light on an often-discussed dental research question that cannot be easily investigated using *in vivo* methods.

## Materials and methods

### Computer model

**FIGURE 1:**
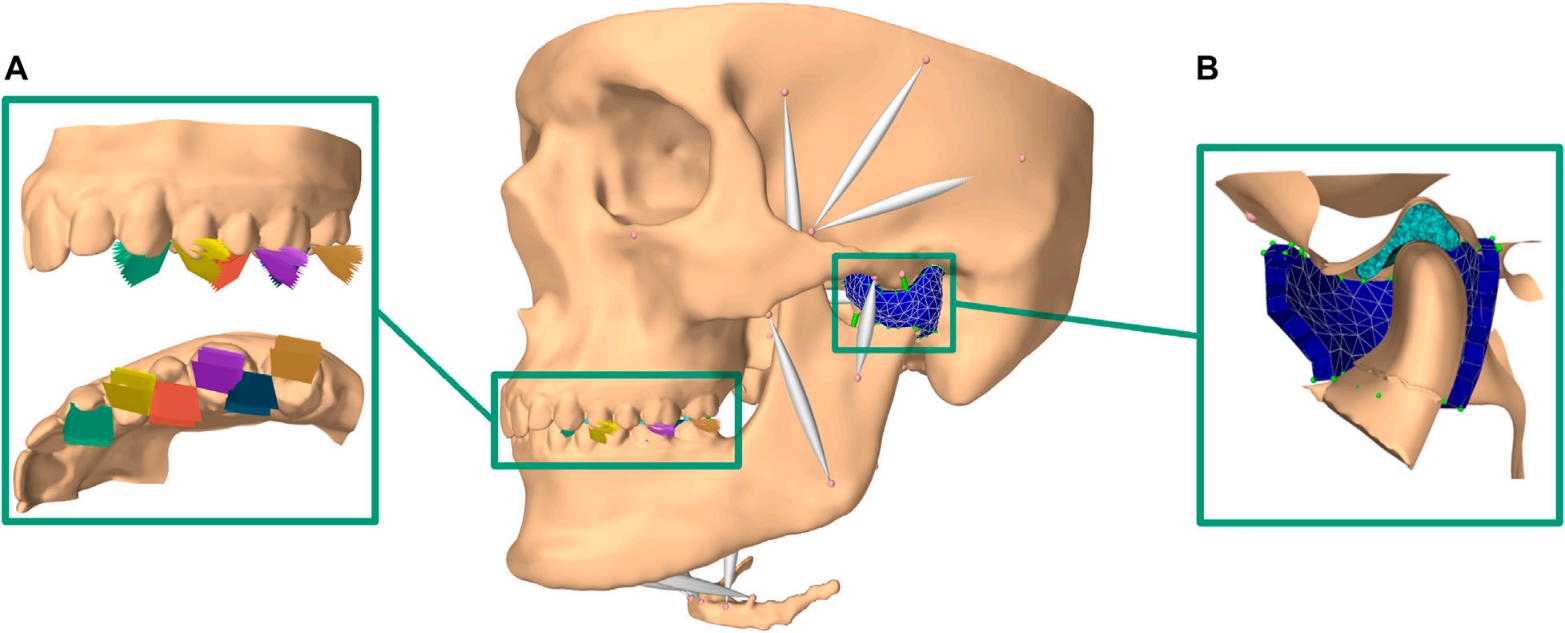
Overview of modeling set-up; **(A)** bruxing positions and inclinations included (brown: LT on second molar, blue: MT on second molar; violet: LT on first molar; red: MT on first molar; yellow: LT on first premolar; green: MT on first premolar)); **(B)** cut through the improved TMJ model using a FE TMJ capsule.

The *in silico* model of the masticatory region is based on high-resolution data from a symptom-free, male volunteer. Approval of the institutional review board of the Medical University of Vienna (Nr. 1190/2017) and written informed consent were obtained. Details on image acquisition and model development have been previously published (Sagl et al., 2019b; Sagl et al., 2019c). In summary, one full skull CT scan combined with a full skull MRI scan and high-resolution TMJ MRI scans were segmented and used to create surface meshes of all involved structures as well as muscle paths. The *in silico* model was built using the ArtiSynth modeling toolkit (www.artisynth.org) (Lloyd et al., 2012). Bones were represented as rigid bodies and muscles as Hill-type line actuators (Hill, 1953). The articular cartilage layers were included as elastic contact foundation layers (Blankevoort et al., 1991) and the TMJ discs as FE models, allowing detailed analysis of mechanical TMJ disc loading. A Mooney Rivlin material (C1 = 9 · 10^5^Pa and C2 = 9 · 10^2^Pa), taken from previous literature, was used (Koolstra and Van Eijden, 2005). Model validation as well as a sensitivity analysis were performed earlier (Sagl et al., 2019c). Compared to our previous bruxing simulations we improved the model by adding a representation of each TMJ capsule as a uniform layer of approximately 400 FE wedge elements, combined with inextensible cables representing the TMJ ligaments (Figure 1). A detailed description of the approach can be found in (Sagl et al., 2021).

### Bruxing facet morphology

The upper tooth bruxing facet was simplified using a planar, bilateral constraint. The LT inclination of the plane was altered using the mean values for the lateral guiding facet, with respect to the axis-orbital plane, of the canine, first premolar, first molar and second molar (3.1°, 8.1°, 20.9° and 46.7°) (Kulmer et al., 1999). Additionally, 0° inclination simulations were included to model fully abraded, flat teeth. Furthermore, the bruxing plane was rotated by -10°, -5°, 0°, 5° and 10° in the sagittal plane. This angle represents the anterior-posterior steepness of the tooth cusp. To investigate the effect of the grinding position on the dentition we ran simulations on the first premolar, first molar and second molar. Since no literature on the mean steepness of MT interferences could be found, the same angles as for the LT simulations were used, just mirroring the lateral guidance angle. See Table 1 for an overview of all variables and their potential states.

**TABLE 1:** Overview of variables included in the investigation.

Variable	Included values
Bruxing motion	Laterotrusive direction, mediotrusive direction
Tooth position	First premolar, first molar, second molar
Lateral inclination	0°, 3.1°, 8.1°, 20.9°, 46.7°
Sagittal tilt	−10°, −5°, 0°, 5°, 10°
Bruxing force	150, 200, 250N

### Bruxing motion and force application

We strived to solve the problems connected to detailed EMG acquisition of the masticatory muscles by using our forward-dynamics tracking approach that allows the use of motion as well as reaction force targets. A detailed explanation and theoretical description of the optimization method can be found in (Sagl et al., 2019a).

In short, at each time step an optimization problem was solved with incorporated kinematics and kinetic goals:

$$\Phi_{v}\;\left(\text{a}\right)\text{=}\frac{\text{1}}{\text{2}}\;{\left\|\bar{v}-H_{v}a\right\|}^{\text{2}}\Phi_{c}\left(\text{a}\right)=\frac{\text{1}}{\text{2}}{\left\|\bar{c}-H_{c}a\right\|}^{2}$$ (1)

where $\Phi_{v}$ and $\Phi_{c}$ are the quadratic optimization goals minimizing the difference between a movement and/or reaction force (i.e. bruxing force) goal, $\bar{v}$ or $\bar{c}$ , and the movement and/or force resulting from a set of muscle activations, represented by $H_{v}a$ and $H_{c}a$ .

The optimal, defined with respect to the optimization function, muscle activation vector a is computed using the optimization equation:

$$\underset{\text{a}}{\text{min}}w_{v}\Phi_{v}\left(a\right)+w_{c}\Phi_{c}\left(a\right)+\frac{w_{a}}{2}a^{T}a+\frac{w_{d}}{2}{\left\|a_{i-1}-a\right\|}^{2}\;\;\mathrm{subject}\;\mathrm{to}\;0\leq a\leq 1$$ (2)

where, $w_{v}$ , $w_{c}$ , $w_{a}$ and $w_{d}$ are the weights for each of the cost term (typically $w_{\text{d}}<w_{a}\ll w_{v},\;w_{c}$ ). The l2 norm regularization term $\frac{w_{a}}{2}{\text{a}}^{T}\text{a}$ is added to solve the muscle load sharing problem. To prevent the simulation from oscillating between two potentially equally feasible states, caused by the larger number of muscles in the small area, a damping term was added, penalizing fast changes in muscle activation patterns. The weights of the regularization term and damping term were set to 0.00025 and 0.00005, respectively.

Tooth contact was always located on the left side of the dentition for both LT and MT simulations. Consequently, the results report the left side as contact side and the right side as non-contact side. The current work focusses on the tooth grinding aspect of bruxism. LT as well as MT bruxing movements with a movement goal of 3 mm excursion along the wear facet were simulated. A goal of 3 mm excursion for all simulations was chosen to keep results comparable and since it lies in the range of mean excursions for the investigated teeth (Kulmer et al., 1999). A second, static movement goal was added at the condyle to penalize non-physiological separation of the TMJ structures. The LT or MT movement goal was weighted with 1 and the static goal was assigned weights of 1 for the medio-lateral, 0.75 for the anterior-posterior and 0.75 for the inferior-superior directions. A time step of 0.001s was used. Bruxing force was applied first, without movement, increasing for 0.05 at the start and was also reduced statically at the end of the simulation for the same amount of time. The bruxing motion itself was simulated over a 0.4s timeframe. Three levels of bruxing force (150N, 200N, 250N) were included in our investigation. These values were chosen to lie in the range of expected tooth grinding force values (Nishigawa et al., 2001) and should allow the majority of grinding simulations to compute successfully. Overall, a total of 450 simulations was computed.

### Reported variables

We report mean von Mises stress over the 200 nodes with the highest stress for the contact side and non-contact side TMJ disc, mean muscle excitation patterns as well as their correlation with TMJ disc loading. The mean stress of the 200 maximally loaded nodes was chosen, because it represents the region of maximum stress of the disc without being sensitive to single nodes with extremely high stresses. This approach allows us to investigate the maximal loading on the TMJ disc while keeping our results comparable and stable.

## Results

### Simulation overview

Of the 450 simulations computed 398 simulations finished successfully, with the other simulations failing due to overloading on one of the TMJ discs. As expected, more simulations failed for higher bruxing force targets. Additionally, an increased number of simulations failed using the first premolar compared to other tooth positions (Supplementary Figure S1 and Supplementary Figure S2).

### TMJ disc stress

**FIGURE 2:**
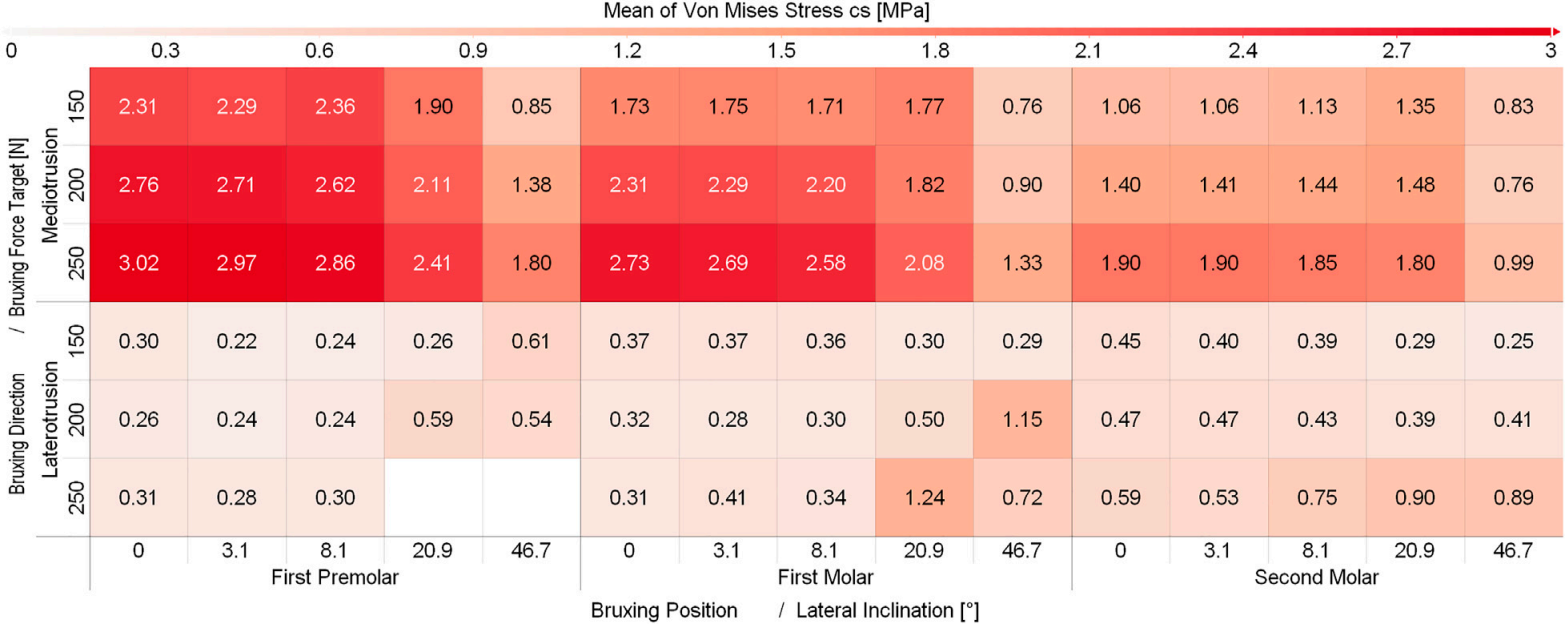
Mean von Mises Stress of the 200 highest loaded nodes on the contact side disc for different simulations.

**FIGURE 3:**
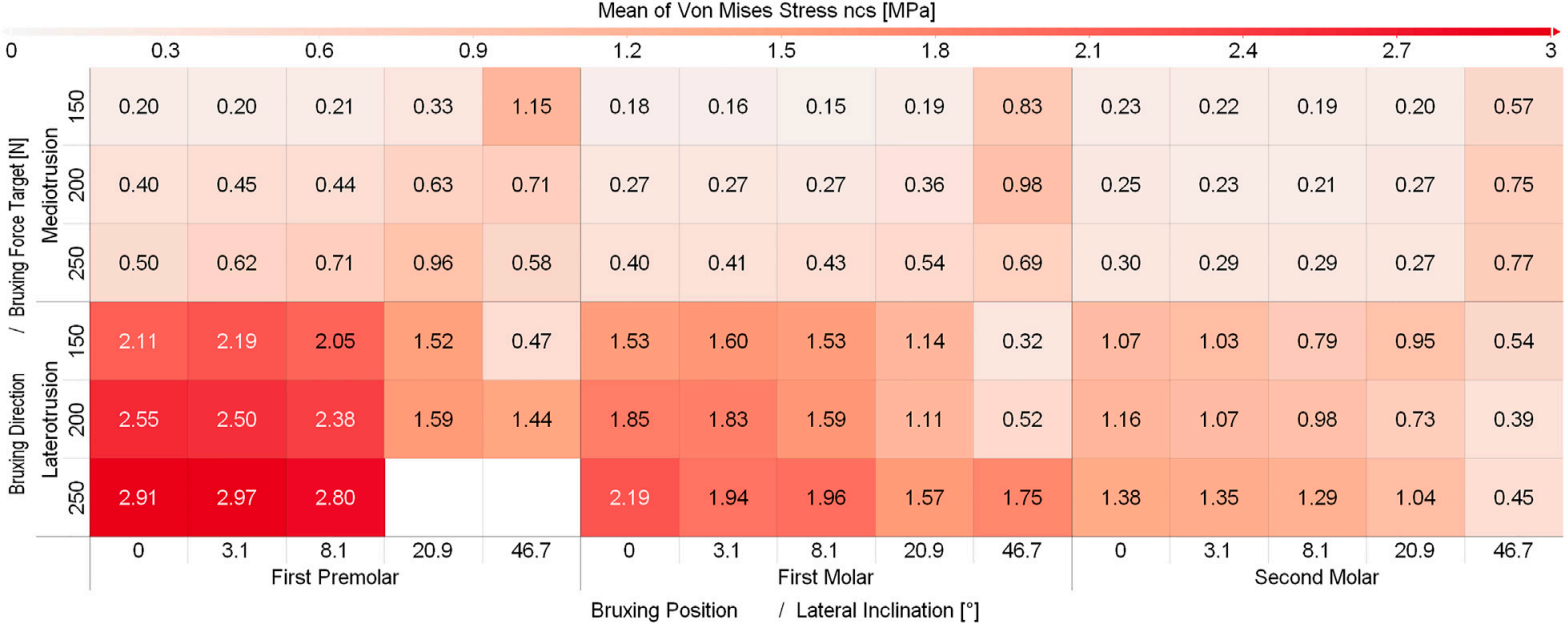
Mean von Mises Stress of the 200 highest loaded nodes on the non-contact side disc for different simulation.

While some differences between different sagittal tilt angles could be seen, no clear trends with respect to TMJ disc loading could be identified (Supplementary Figure S1 and Supplementary Figure S2). Consequently and to increase readability of figures, all results were presented using the mean over all sagittal tilt angles computed for a specific position, lateral inclination, force and movement goal (e.g., mean over all sagittal tilt angles for the von Mises stress of the left TMJ disc for a MT movement on the first molar with a lateral inclination of 8.1° using 250N bruxing force). Simulation results for all simulation groups can be seen in Figure 2 for the left (contact side) and Figure 3 for the right (non-contact side) TMJ disc. The highest von Mises stress on the contact side disc was reported with 3.02 MPa for MT bruxing with a 250N force target and 0° lateral inclination while grinding on the first premolar. On the non-contact side disc, the highest stress was reported with 2.91 MPa for the same condition, but during a LT movement. Generally speaking, higher loading was seen for MT movements on the contact side and LT movements on the non-contact side (Figure 2 and Figure 3).

### Muscle excitations

**FIGURE 4:**
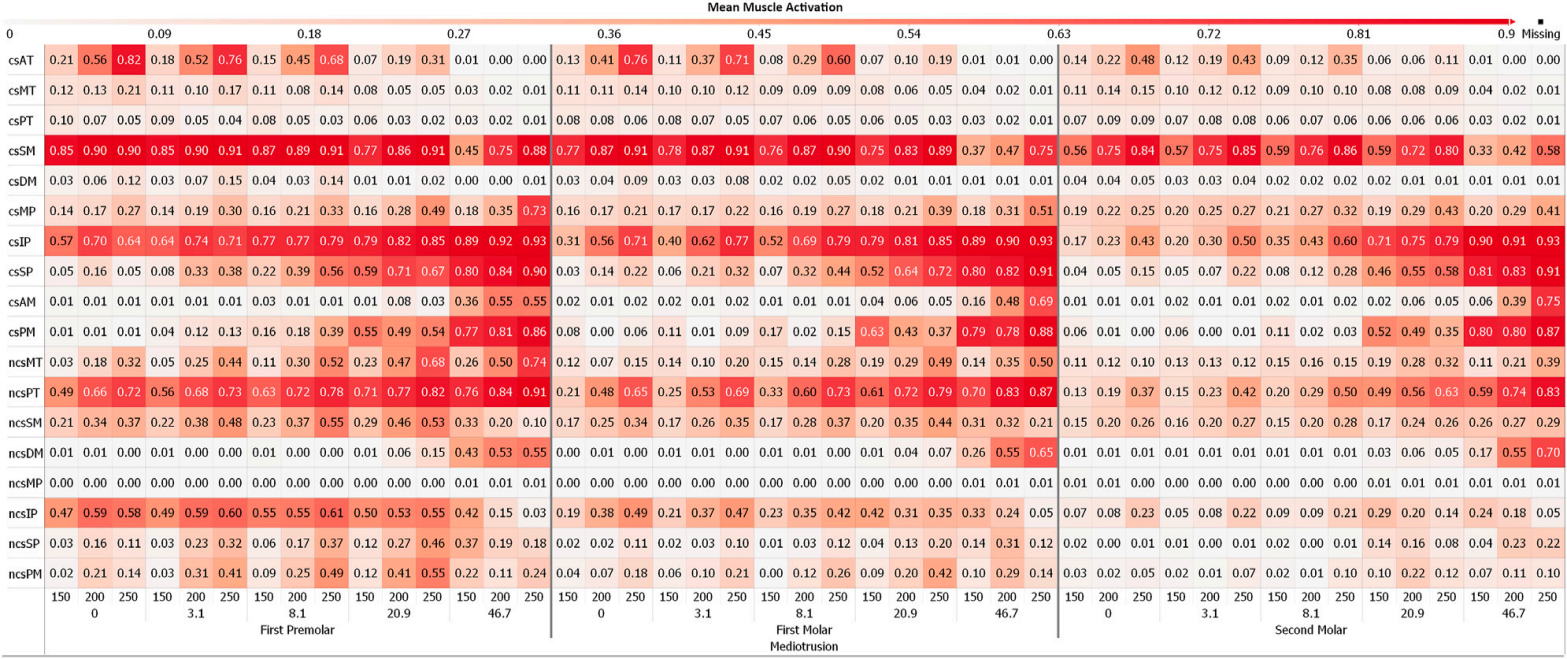
Mean muscle activation for all muscles with an activation >20% for mediotrusive movements; 0, 3.1, 8,1, 20.9 and 46.7 are tooth facet inclinations in degree; 150, 200 and 250 are the bruxing force levels in Newton; contact side (cs) and non-contact side (ncs); AT, anterior part of temporalis; MT, medial part of temporalis; PT, posterior part of temporalis; SM, superficial head of the masseter; DM, deep head of the masseter; MP, medial pterygoid; IP, inferior head of lateral pterygoid; SP, superior head of lateral pterygoid; AM, anterior mylohyoid; PM, posterior mylohyoid.

**FIGURE 5:**
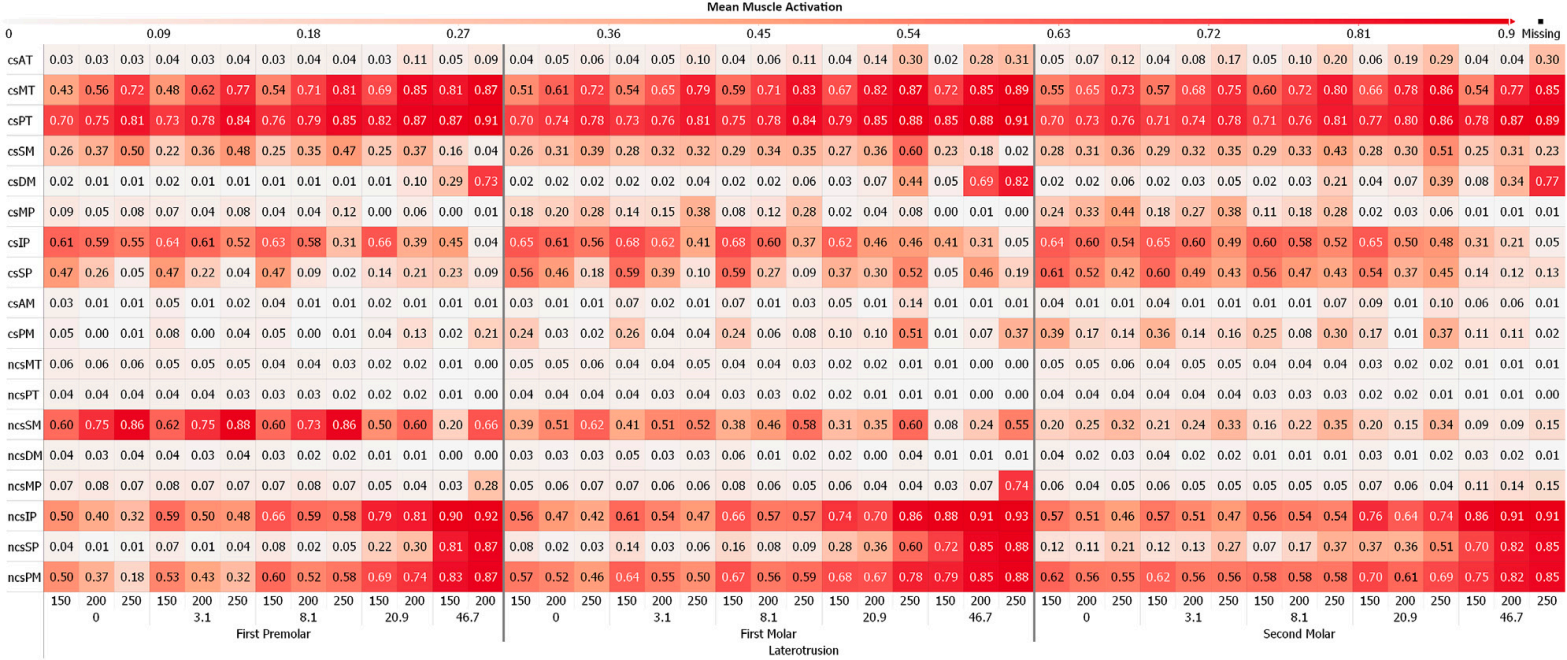
Mean muscle activation for all muscles with an activation >20% for laterotrusive movements; 0, 3.1, 8,1, 20.9 and 46.7 are tooth facet inclinations in degree; 150, 200 and 250 are the bruxing force levels in Newton; contact side (cs) and non-contact side (ncs); AT, anterior part of temporalis; MT, medial part of temporalis; PT, posterior part of temporalis; SM, superficial head of the masseter; DM, deep head of the masseter; MP, medial pterygoid; IP, inferior head of lateral pterygoid; SP, superior head of lateral pterygoid; AM, anterior mylohyoid; PM, posterior mylohyoid.

Figures 4, 5 depict the mean muscle activations, over the course of the simulation, averaged over all sagittal tilt angles for mediotrusive and laterotrusive movements. LT simulations facilitate activation of the medial and posterior temporalis, superficial masseter and lateral pterygoid on the contact side as well as lateral pterygoid, superficial masseter on the non-contact side. Contact side temporalis and non-contact side lateral pterygoid activation increases with increased inclination. For MT simulations, mostly activation of contact side superficial masseter, lateral pterygoid and mylohyoid muscle can be seen. Non-contact side activation can be seen for the lateral pterygoid and superficial masseter as well as increasing activation of the posterior temporalis with increasing inclination.

**FIGURE 6:**
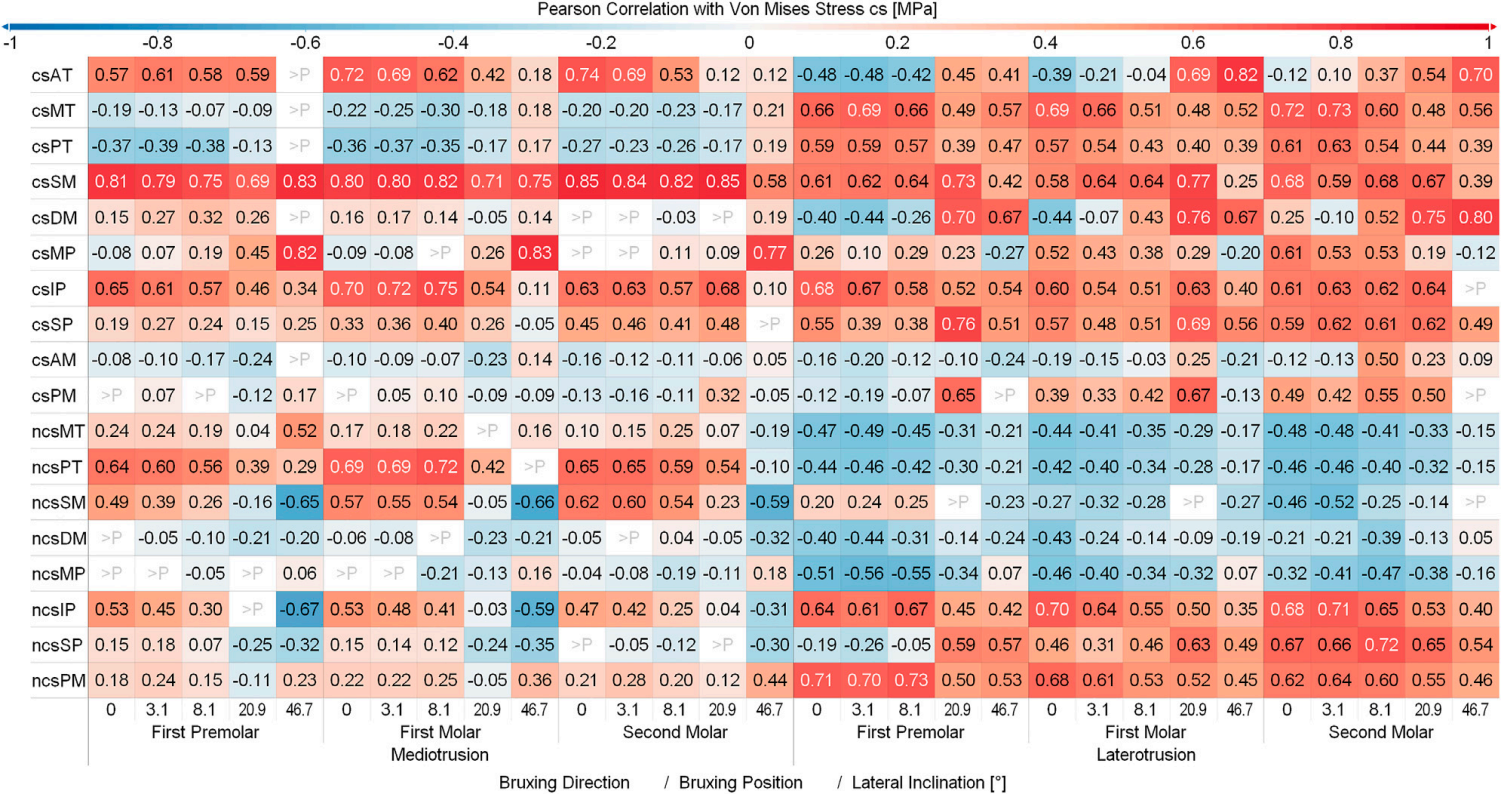
Correlation between muscles and contact side disc von Mises stress. Y-axis shows muscles with a mean activation >20%; contact side (cs) and non-contact side (ncs); AT, anterior part of temporalis; MT, medial part of temporalis; PT, posterior part of temporalis; SM, superficial head of the masseter; DM, deep head of the masseter; MP, medial pterygoid; IP, inferior head of lateral pterygoid; SP, superior head of lateral pterygoid; AM, anterior mylohyoid; PM, posterior mylohyoid.

**FIGURE 7:**
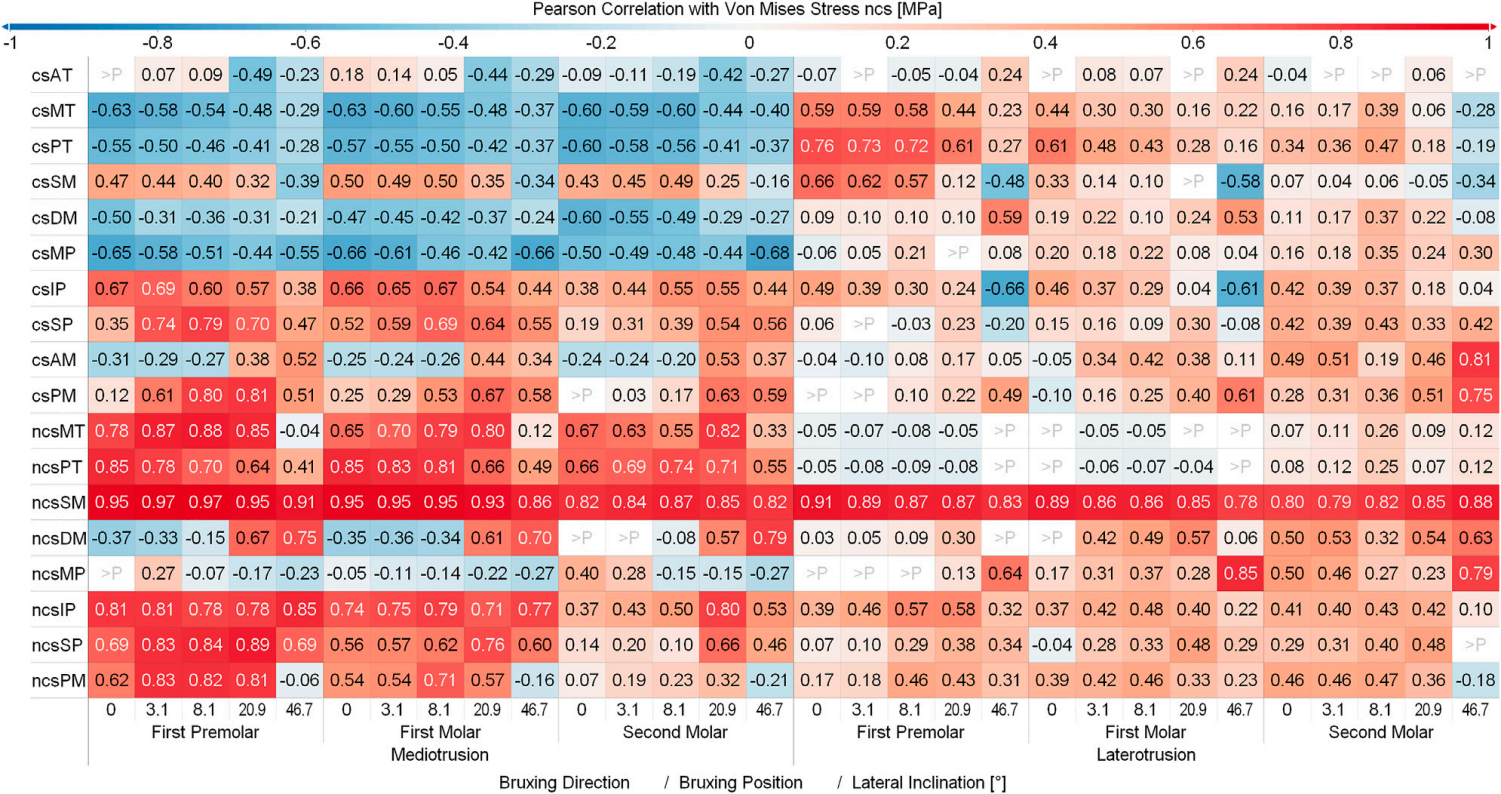
Correlation between muscles and non-contact side disc von Mises stress. Y-axis shows muscles with a mean activation >20%; contact (cs) and non-contact side (ncs); AT, anterior part of temporalis; MT, medial part of temporalis; PT, posterior part of temporalis; SM, superficial head of the masseter; DM, deep head of the masseter; MP, medial pterygoid; IP, inferior head of lateral pterygoid; SP, superior head of lateral pterygoid; AM, anterior mylohyoid; PM, posterior mylohyoid.

Figure 6 and Figure 7 show the Pearson Correlation (computed for *p* < 0.05) between the activation of each of the masticatory muscles and TMJ disc loading of the contact side and non-contact side TMJ disc respectively. To keep the figure more readable the average over all tilt angles and bruxing force levels was reported. The non-contact side and contact side superficial masseter have the highest positive correlation with TMJ loading for the respective TMJ discs. Other large positive correlations for non-contact side TMJ disc stress during MT can be seen for both heads of the non-contact side lateral pterygoid, medial and posterior compartments of the temporalis, posterior mylohyoid as well as the contact side lateral pterygoid muscle for MT movements. For the contact side disc correlations are generally not as clear with many muscles showing some correlation with loading, especially for LT movements. Negative correlations were not as strong, with largest values for the contact side medial pterygoid (max. -0.68) and medial compartment of the temporal muscle (max. -0.63) during MT for the non-contact side disc. There seem to be no clear trends in negative correlations for both discs, with some negative correlations for the closing muscles of the opposite side (non-contact side muscles for contact side disc and vice versa).

## Discussion

In this paper we present a thorough investigation of the effect of differences in tooth cusp morphology and movement direction on TMJ disc mechanical loading during bruxing. The combination of a detailed *in silico* model with a forward-dynamics tracking algorithm additionally enables the investigation of changes in muscle recruitment caused by these variables. Overall, a total of 450 simulations have been computed as part of this study. Our results strengthen some findings from previous studies and give new insights into the effect of grinding motion as well as the correlation between specific muscles and TMJ loading. Overall, the study strived to increase the understanding of the effect of various variables on TMJ loading during bruxism and could help broaden the understanding of the disease’s connection to TMD.

### Bruxing force

In previous work the maximal bruxing force was defined manually for each of the 12 specific simulation set-ups (Sagl et al., 2022). Since our new investigation included a total of 450 simulations manual examination of the maximal force was not feasible. Instead, we decided to use three different force levels, 150N, 200N and 250N for all set-ups. These values lie in the range of 200 ± 127N reported in previous literature for molar grinding (Nishigawa et al., 2001). Three different force levels were used since steeper lateral guidance angles generally prohibit large grinding forces, consequently we wanted to include lower levels of bruxing force that still enabled a successful completion of such simulations (Sagl et al., 2022). A larger value than 250N would fail to compute for most simulations, hence we decided against including even higher bruxing forces. Consequently, some of the molar simulation cases are most likely simulating sub-maximal bruxing forces.

### Mediotrusive vs laterotrusive contact

Additionally, differences in occlusion can lead to different tooth contacts and thus change tooth grinding patterns (Onodera et al., 2006). More specifically, our study focused on the investigation of potential differences between MT and LT guidance. While the topic of MT guidance is still controversially discussed in literature, generally anterior LT guidance seems to be preferred, when redesigning occlusal contacts (Walton and Layton, 2021). Due to the changes in the direction of movement, differences in muscle excitation patterns can be seen. The contact side inferior pterygoid, superficial masseter and non-contact side posterior temporalis are the muscles with the highest activation over all MT simulations. Moreover, contact side superior lateral pterygoid and mylohyoid activation seem to ramp up with increased guidance inclination. These muscles are used to pull the condyle forwards and create more mouth opening, which are both needed with higher lateral guiding facet inclinations (Murray et al., 2007). For LT, high activation of the contact side medial and posterior temporalis can be seen as well as non-contact side inferior lateral pterygoid, mylohyoid and superficial masseter activation. The contact side lateral pterygoid seems to be strongly activated for submaximal grinding tasks. The differences in muscle activations lead to higher TMJ disc stress on the contact side for MT and the non-contact side for LT movements. We think that these increased loads are mainly caused by the anterior movement of the respective condyle and disc (contact side for MT and non-contact side for LT), in combination with bruxing force application. Overall, higher loading can be seen during MT bruxing tasks, suggesting that LT guidance might have a protective effect for the TMJ.

### Facet inclination

We previously reported a potential decrease in TMJ loading with increased lateral guidance inclination during bruxing (Sagl et al., 2022). The current study expanded this investigation, by including 5 different levels of inclination ranging from 0° to 46.7°. The chosen values represent the mean lateral inclination of the canine, first premolar, first and second molar (Kulmer et al., 1999). The results of our more thorough investigation agree well with the previous observation and show a clear trend towards decreased TMJ loading with increased lateral guidance inclination. Moreover, changes in muscle excitation patterns for different inclination values agree well with our previous investigation and clinical investigations of changes to the inclination of the LT guidance (Huang et al., 2006). Huang et al. report a decrease of contact side anterior and posterior temporalis activation with increasing steepness, which was not seen in our study (Huang et al., 2006). We think that this is caused by the submaximal nature of our grinding tasks and would expect to see the same decrease when computing the maximally possible bruxing force for each inclination. Their study mostly focussed on the activation of changes in lateral pterygoid activation, which agree well with our results. The contralateral medial pterygoid was only recruited for higher inclination simulations. We could not find any previously reported data on muscle excitations during MT grinding. Overall, our simulations show a clear trend towards decreased TMJ loading with an increase in lateral facet inclination, especially on the disc which is protruded during the movement. This fact holds true over all included positions, movement directions and force levels and clearly strengthens the evidence behind the effect of lateral facet inclination on TMJ loading.

### Grinding position

Muscle activation patterns seem to only differ slightly between bruxing positions. A trend towards increased activation of the non-contact side, for LT, or contacts side, for MT, superficial masseter for premolar grinding can be seen. Consistent with our previous studies, an increase of TMJ loading for both discs can be seen for more anterior bruxing positions, with the highest loading reported for premolar grinding. Potentially, this increase might be caused by the longer distance between the TMJ and a more anterior bruxing position, which leads to a longer moment arm. While initially this relationship was rather weak in our previous study, our results including two additional positions strengthen the hypothesis that an effect of bruxing position on TMJ loading exists. Nevertheless, the effect of facet inclination seems to be much stronger and since anterior teeth are generally steeper than posterior teeth, this positional effect might be less distinct in actual patients.

### Facet tilt

As an additional variable our investigation included the sagittal facet steepness. Since no detailed literature reports on the mean value for this tilt exist, we decided to vary it by ±10° from the original angle estimated from the unaltered dentition of the volunteer the model was based on. This should give us a range broad enough to include most of the common sagittal tilt values. For lateral movements, we could not see a clear trend towards specific tilt angles and consequently its effect was not further studied in detail. This might be the case since the sagittal tilt is not in the main direction of mandibular movement during lateral bruxing. Consequently, this variable might have a larger influence when simulating anterior-posterior bruxing.

### Differences to previous studies

While our results overall reinforce our previous findings on muscle activation during laterotrusive bruxing, some differences in muscle activation pattern can be observed. While we do see the same increase of the muscle activation of the contact side lateral pterygoid with increased inclination, the overall activation level is higher during LT (Sagl et al., 2022). This is most likely caused by the improved TMJ capsule model used for this study. Previously, the capsule was only modeled using 4 axial springs which restricted the movement of the disc relative to the condyle but did not add any of the inertia caused by the surrounding tissue. The new FE representation now leads to a more stable and more inert model, which might lead to some differences in muscle excitation. Moreover, contact side medial and posterior temporalis activation seems to increase instead of decrease with increased lateral inclination. As described above, this might be caused by the combination of the increased model stability and the submaximal nature of our bruxing force goals. In our previous study, we were able to create maximum forces of 287N on the first molar for 8.1° and 170N for 46.7° lateral inclination during a LT movement. The current study only included a maximum grinding force of 250N, which due to the increased stability of our new approach, was successfully simulated for both inclinations. Individually increasing the bruxing force to the maximum force possible would most likely lead to the same trend in temporal muscle activation but is not a feasible approach for a total of 450 simulations. Nevertheless, simulation results overall agree well with previous literature on muscle activation and TMJ disc loading during LT movements. To the best of our knowledge no comparable data exist for MT movements.

### Clinical implications

By cautiously analysing our *in silico* results some implications for clinicians can be found. While the position of tooth contact during bruxing seems to have some effect, the main differences can be seen with respect to the lateral guidance. Relatively steep lateral guidance surfaces seem to have a protective effect on TMJ loading. Since teeth cannot be made arbitrarily steep, due to loss of other important functions like force creation during chewing (Drake et al., 2019), our results suggest that a lateral canine guidance (using the naturally “steepest” tooth) might be beneficial. Moreover, we do report higher overall TMJ loading during MT bruxing movements. While further investigations are needed to investigate if this difference is large enough to justify proactive occlusal alterations it might suggest that MT contacts should be avoided when designing new occlusal surfaces as part of a prosthodontic restorations. The current results should not be seen as clear clinical evidence, but as motivations for future studies to verify these implications using clinical trials.

### Limitations and future work

The current *in silico* study contains some assumptions and limitations. First, while we computed a large number of simulations altering morphological variables of the bruxing tooth cusp, the TMJ structures are based on a single symptom-free volunteer. Furthermore, there exists a sizable ambiguity with regards to the mechanical properties of the articular, disc and muscle tissues. Additionally, the forward-dynamics tracking approach used to predict muscle patterns includes some assumptions to solve the redundancies related to muscle force sharing between agonist muscles. The presented results simulate bruxing with contact on a single tooth which simplifies the often complex progression of tooth abrasion. Consequently, an interesting step would be to expand our optimization approach to computed results for group bruxing on multiple teeth. As described previously, our approach facilitates a motion target that was not directly measured (Sagl et al., 2022). This is caused by the fact that to measure actual movements for each simulated tooth morphology we would have to grind down teeth of a human subject, which is not a feasible approach while keeping the vertical dimension from a patient-safety point of view. For lateral bruxing movements, the problem is diminished by the relatively small movement which is mostly governed by the morphology of the upper jaw tooth. Moreover, the potentially disadvantageous effect of prescribing the kinematics directly was lessened by using mandibular motion as an optimization goal and consequently allowing slight deviation from the target motion by minimizing the error over all optimization terms of the bruxing task. The amount of lateral excursion as well as the inclination values were taken from previous literature for LT values (Kulmer et al., 1999). Unfortunately, no values for MT motions could be found. We decided to use the values derived for LT motions, since one would expect mandibular motion to be comparable and the facet inclination for MT contact to be at least as high as the values for LT motion, since otherwise LT contact would occur. Moreover, the use of the same values for both directions make the computed TMJ disc stress results more readily comparable. Another interesting topic, which was not included in this study, is the relation between muscle activation, fatigue and pain. Previous literature, investigating haemodynamics during clenching, suggests that low-force, long-duration clenching is more harmful to the muscle than high-force, short clenching spurts (Satokawa et al., 2020). While our current study focused on TMJ loading, the inclusion of muscle and TMJ metabolisms could be a very interesting avenue for future research.

Lastly, it has to be stated that, while our *in silico* modeling is a valuable means for the comparative investigation of mechanical loading, enough uncertainty in material properties and modeling approaches exist that results should be interpreted cautiously. Because no *in vivo* measurements of tissue stiffnesses exist, and the patient-specific nature of biomechanical approaches suggest rather large anatomical variations, the presented values should not be seen as clinical thresholds (Tsouknidas et al., 2013).

## Conclusion

In conclusion, our study proposes an innovative, forwards-dynamic tracking approach to investigate the effect of bruxing motion and tooth morphology on TMJ loading. In total, 450 simulations were conducted and analyzed to predict muscle activations and TMJ loads for a range of occlusal conditions. The results corroborate previous findings that increased lateral inclination leads to a decrease in mechanical TMJ loading. While the effect of bruxing facet position seems to be smaller, nevertheless more anteriorly located tooth contact leads to higher TMJ von Mises stress. MT movement overall leads to higher mechanical loading of the joint compared to LT movements. The presented results strengthen the evidence that tooth morphology influences TMJ loading. We hope this modeling study will encourage future clinical investigations into the connection between tooth shape and masticatory biomechanics.

## Data Availability

The raw data supporting the conclusions of this article will be made available by the authors, without undue reservation.
