# Supplementary figures and images for: The effect of tooth cusp morphology and grinding direction on TMJ loading during bruxism
Source: Front Physiol. 2022 Sep 15;13:964930. doi: 10.3389/fphys.2022.964930 (PMC9521318; doi:10.3389/fphys.2022.964930)

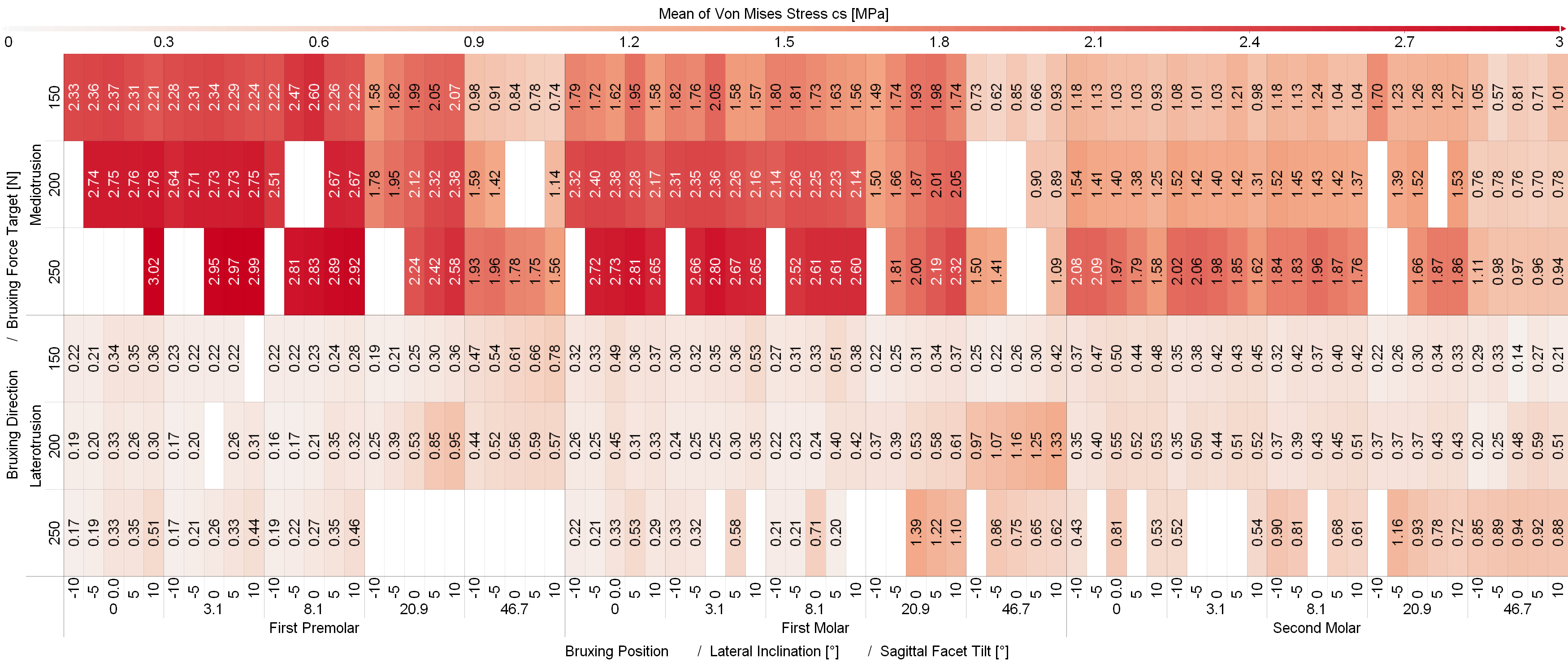

Supplement: Supplementary file 1 [file Image1.TIFF]

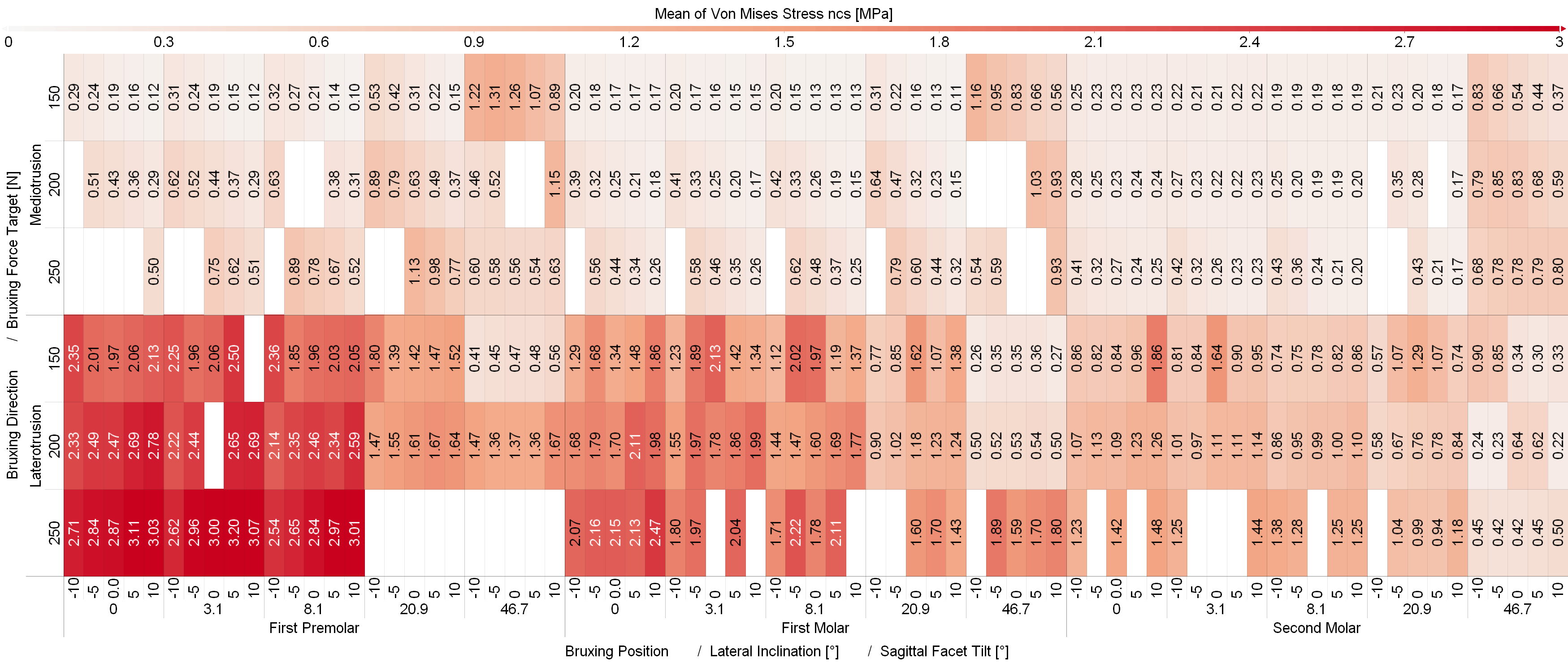

Supplement: Supplementary file 2 [file Image2.TIFF]
